# Beneficial and Detrimental Effects of Regulatory T Cells in Neurotropic Virus Infections

**DOI:** 10.3390/ijms21051705

**Published:** 2020-03-02

**Authors:** Malgorzata Ciurkiewicz, Vanessa Herder, Andreas Beineke

**Affiliations:** 1 Department of Pathology, University of Veterinary Medicine Hannover, Bünteweg 17, D-30559 Hannover, Germany; 2 Center for Systems Neuroscience Hannover, University of Veterinary Medicine, D-30539 Hannover, Germany; vanessa.herder@tiho-hannover.de; 3 MRC—University of Glasgow, Centre for Virus Research, 464 Bearsden Road, Glasgow G61 1QH, Scotland, UK

**Keywords:** regulatory T cells, Foxp3, central nervous system, neurotropic viruses, neuroinflammation, animal models, demyelination

## Abstract

Neurotropic viruses infect the central nervous system (CNS) and cause acute or chronic neurologic disabilities. Regulatory T cells (Treg) play a critical role for immune homeostasis, but may inhibit pathogen-specific immunity in infectious disorders. The present review summarizes the current knowledge about Treg in human CNS infections and their animal models. Besides dampening pathogen-induced immunopathology, Treg have the ability to facilitate protective responses by supporting effector T cell trafficking to the infection site and the development of resident memory T cells. Moreover, Treg can reduce virus replication by inducing apoptosis of infected macrophages and attenuate neurotoxic astrogliosis and pro-inflammatory microglial responses. By contrast, detrimental effects of Treg are caused by suppression of antiviral immunity, allowing for virus persistence and latency. Opposing disease outcomes following Treg manipulation in different models might be attributed to differences in technique and timing of intervention, infection route, genetic background, and the host’s age. In addition, mouse models of virus-induced demyelination revealed that Treg are able to reduce autoimmunity and immune-mediated CNS damage in a disease phase-dependent manner. Understanding the unique properties of Treg and their complex interplay with effector cells represents a prerequisite for the development of new therapeutic approaches in neurotropic virus infections.

## 1. Introduction

A variety of viruses are able to infect the central nervous system (CNS) and contribute to neurologic diseases in humans and animals worldwide. Although most viral infections are self-limiting and asymptomatic in immunocompetent hosts, some neurotropic viruses can cause acute and fatal or severely debilitating inflammation. Moreover, virus persistence or long-term neurologic disabilities, as well as an increased risk of developing epilepsy, can be observed in patients surviving acute viral encephalitis [1,2,3]. CNS lesion development and disease outcome depends on host factors (e.g., age, gender, immunogenetics) and viral properties. Virus infection of the CNS typically leads to lymphocytic inflammation, glial activation, and tissue damage, including necrosis and demyelination (Table 1). Besides direct virus-induced cytopathology, several neurologic disorders are caused by excessive antiviral immune responses [3]. Certain pathogens, including herpesviruses, are also suspected triggers of autoimmune diseases, such as multiple sclerosis (MS), anti-N-methyl-D-aspartate (NMDA) receptor encephalitis, or Guillain.

Barré syndrome occurs in predisposed individuals [31,32]. Thus, despite the need for protection against viral invasion and replication, the extent of the immune response in the CNS needs to be carefully regulated to prevent bystander tissue damage and immunopathology. Regulatory T cells (Treg), a cluster of differentiation (CD)4^+^ T cell subset with immunosuppressive and immunomodulatory functions, are principle regulators of immune reactions and mediators of peripheral tolerance. They contribute to tissue homeostasis under steady state conditions and regulate immune responses in inflammatory diseases [33,34]. Immunomodulation represents a promising new application for treating autoimmune and infectious diseases, including neurologic disorders. Mechanisms to enhance regulatory T cell function in order to prevent pathogen-induced, immune-mediated tissue damage as well as approaches to reduce their function to strengthen protective immunity and vaccination immunogenicity have been proposed [35,36,37]. The present review summarizes the current knowledge on Treg involvement in human CNS infections and the effects of Treg manipulation in experimental models of human neutrotropic virus infection and virus-induced demyelination.

## 2. Biology of Regulatory T cells

**Figure 1:**
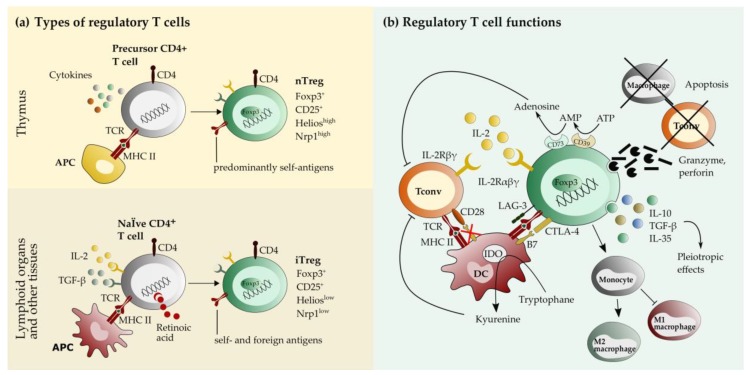
Types and functions of regulatory T cells (Treg). (**a**) There are two main types of Treg in humans and mice. Natural Treg (nTreg) are generated in the thymus from precursor T cells recognizing self-antigens presented by thymic antigen presenting cells (APC) via major histocompatibility complex (MHC) II molecules. The differentiation to the Treg phenotype is further influenced by local cytokines. nTreg express high levels of Helios and neuropilin-1 (Nrp1). Treg can also be generated de novo from naïve conventional cluster of differentiation (CD)4^+^ T cells in extrathymic tissues in the presence of interleukin (IL)-2, transforming growth factor (TGF)-β, and retinoic acid following T cell receptor (TCR) engagement. These induced Treg (iTreg) express low levels of Helios and Nrp1. Both Treg types are characterized by the expression of the transcription factor forkhead box protein P3 (Foxp3) and CD25. (**b**) Treg exert their suppressive function by various cell contact-dependent and -independent mechanisms. These include cytotoxic T-lymphocyte-associated protein 4 (CTLA-4)-dependant suppression of B7-mediated co-stimulation of conventional T cells (Tconv), Lymphocyte activation gene 3 (LAG-3)-mediated suppression of dendritic cell (DC) maturation, IL-2 deprivation of other T cells through expression of high-affinity IL-2 receptors (IL2R), and generation of the immunosuppressive nucleotide adenosine by the ectoenzymes CD39 and CD73. Moreover, Treg secrete the anti-inflammatory cytokines IL-10, TGF-β, and IL-35, and induce apoptosis of inflammatory cells through granzyme/perforin secretion. Treg also shift macrophage polarization from M1 to the M2 type and induce the activity of the immunosuppressive enzyme indoleamine 2,3-dioxygenase (IDO) in DCs.

Prevention of immunopathology in the course of inflammation and maintenance of self-tolerance requires mechanisms that dampen deleterious immune responses. Several cell types with regulatory functions have been described, including CD4^+^CD25^+^forkhead box protein P3 (Foxp3)^+^ regulatory T cells (Treg), type 1 regulatory T cells (Tr1 cells), invariant natural killer T cells, double negative CD3^+^ helper cells, γδ-T cells, CD8^+^Foxp3^+^ T cells, regulatory B cells, and myeloid suppressor cells. Among them, Treg are the most-studied and well-characterized cell type, and their paramount importance for immune homeostasis has been amply demonstrated [38,39,40,41]. Defects in the gene coding for the Treg-specific transcription factor forkhead box protein P3 (Foxp3) lead to an aggressive and fatal autoimmune disorder known as immune dysregulation, polyendocrinopathy, enteropathy, X-linked syndrome (IPEX) in humans and a comparable systemic lymphoproliferative disease in scurfy mutant mice [42,43]. Besides their role in peripheral tolerance to the self, Treg are also involved in the suppression of excessive immune responses evoked by commensal microbiota and invading pathogens [44,45]. There are two main types of Treg: natural Treg (nTreg) are generated in the thymus, whereas induced Treg (iTreg) arise de novo from naïve CD4^+^ T cells in the periphery upon Foxp3 induction mediated by interleukin (IL)-2 and transforming growth factor (TGF)-β signaling (Figure 1a) [46,47]. Treg exert inhibitory functions on a variety of immune cells, including T and B cells, natural killer cells, and cells of the innate immune system via cell contact-dependent and -independent mechanisms (Figure 1b). For instance, Treg show a high expression of cytotoxic T-lymphocyte-associated protein 4 (CTLA-4), which competes with CD28 for the co-stimulatory molecules B7-1 and B7-2 on antigen-presenting cells and thereby prevents T cell priming. Treg require IL-2 derived from other cell sources as a growth and survival factor and constitutively express CD25, which is a part of high affinity IL-2 receptors [48,49,50]. Owing to that, they can deprive other T cells of IL-2, which suppresses their activation and effector function [51]. Interestingly, although Treg restrict the population of CD8^+^ effector T cells, they promote the formation of memory T cells in viral infections of the CNS (see below). The most important Treg-secreted soluble mediators are the anti-inflammatory cytokines IL-10, IL-35, and TGF-β, which can induce a plethora of immunomodulatory downstream effects on a variety of responsive cell types. Treg also express the ectoenzymes ATP apyrase (CD39) and ecto-5′-adenosine monophosphate (AMP)-nucleotidase (CD73), which generate the immunosuppressive purine nucleoside adenosine. Moreover, Treg are capable of directly killing syngeneic cells such as T cells, monocytes, and dendritic cells through granzyme/perforin and caspase-3-involving pathways [52,53,54,55]. This ability is not only important for the limitation of overwhelming immune responses but can also facilitate virus elimination through the execution of infected macrophages, as was shown in the murine human immuno-deficiency virus (HIV)-encephalitis model (see below). Moreover, there is cumulating evidence that distinct subtypes of Treg exist in certain tissue environments (e.g., skin and CNS) and that Treg in those niches might be equipped with additional, site-specific effector functions and induce responses beyond immune regulation [56,57].

Treg function in inflammatory disorders is commonly investigated by depletion/inactivation or increase of Treg numbers in mice. To that end, two types of transgenic knock-in mice were developed, which express the human or simian diphtheria toxin receptor (DTR) under control of the Foxp3 promoter and allow a specific Treg depletion upon DT injection (Foxp3^DTR^ or depletion of regulatory T cells (DEREG) mice) [58,59]. Moreover, functional inactivation of Treg is possible using antibodies directed against CD25 (clone PC61) [60]. Enhancement of Treg responses is achieved by adoptive transfer of Treg or by expansion of endogenous Treg using IL-2 immune complexes (IL-2C, consisting of recombinant IL-2 and anti-IL-2-antibodies, clone JES6-1), which selectively expand cells expressing CD25 [34,61,62]. The approaches targeting CD25 have the disadvantage of simultaneous inactivation or expansion of other cells transiently expressing CD25 (e.g. activated T cells) [61,63], whereas Foxp3 is highly specific for the Treg population.

## 3. Regulatory T Cells in Neuroinflammation and Neuroprotection

**Figure 2:**
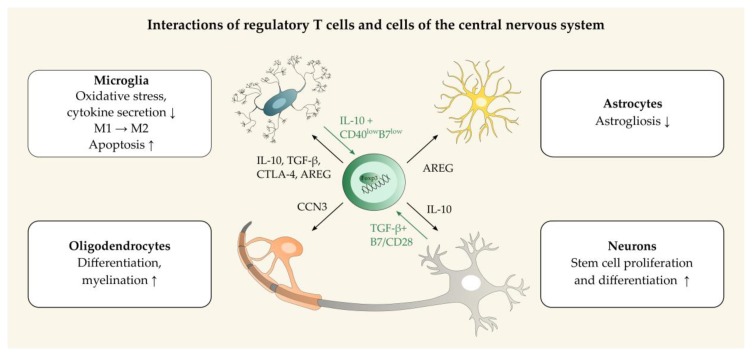
Examples of interactions between regulatory T cells (Treg) and cells of the central nervous system (CNS). Treg influence the function and phenotype of resident CNS cells (black arrows, boxes). For instance, Treg alter microglial responses and suppress astrogliosis via secretion of amphiregulin (AREG) and cytokines, as well as through cell contact-dependent mechanisms. Moreover, Treg are involved in CNS regeneration: Treg-derived cellular network communication factor 3 (CCN3) promotes (re)myelination and differentiation of mature oligodendrocytes in vitro, and IL-10 induces proliferation and differentiation of neuronal stem cells in vivo. Neurons and microglia can induce Foxp3-expression and a regulatory phenotype in CD4^+^ T cells (green arrows) through secretion of TGF-β and B7/CD28 interaction or secretion of IL-10 in combination with low expression of co-stimulatory molecules, respectively. In addition, several factors secreted by glial and neuronal cells promote CNS infiltration of peripheral Treg (not depicted, for details see main text).

Treg play a critical role for immune homeostasis and prevent immunopathology in the brain and spinal cord though interaction with infiltrating immune cells and resident CNS cells (Figure 2). The beneficial impact of Treg functions in the context of autoimmune CNS disorders has been extensively studied in experimental autoimmune encephalomyelitis (EAE), an animal model for human MS. Treg have been shown to suppress encephalitogenic T cells, resulting in sustained self-tolerance [64]. Besides exerting modulatory effects in peripheral lymphoid organs, infiltrating Treg directly influence the local inflammatory milieu in the CNS [65,66]. The choroid plexus is thought to favor the entrance of Treg and T helper (Th)17 cells, whereas the blood–brain barrier may favor the CNS recruitment of Th1 cells and memory T cells [67]. Chemokine ligands (CCL)1 and CCL20 produced by astrocytes and oligodendrocytes initially attract Treg, as shown in ischemic brains [33]. Moreover, CCL22 secretion by activated microglia leads to an enhanced CNS migration of Treg [68,69]. Recently, a distinct cerebral Treg population has been described, which differs from peripheral Treg in the gene expression pattern. Brain Treg express the transcription factor Helios, killer cell lectin-like receptor subfamily G member 1 (KLRG1), and the serotonin receptor 7, as well as high levels of CTLA-4, ST2 (IL-33 receptor subunit), and programmed cell death-1 (PD-1) [33]. It remains undetermined whether these Treg are locally instructed within the brain to adopt tissue-specific properties or whether specific subsets of Treg are selectively recruited to the CNS [57,58]. Treg-expansion in the CNS is driven by IL-2, the alarmin IL-33, and the neurotransmitter serotonin. IL-33 and serotonin are constitutively expressed in the CNS and are able to substitute the role of IL-2 in mediating Treg survival and maintenance, for example under post-inflammatory conditions [33].

CNS-infiltrating Treg inhibit microglial pro-inflammatory responses [70]. As shown by proteome analyses, co-cultivation with Treg modulates microglial activity in vitro, including inhibition of oxidative stress, cell migration, phagocytosis, and cytokine production. Treg are able to induce apoptosis of activated microglia [71,72]. Moreover, Treg induce a neuroprotective M2-phenotype of microglia with reduced production of neurotoxic factors, such as inducible nitric oxide synthase (iNOS) and nitric oxide [70,73,74]. Brain Treg also suppress neurotoxic astrogliosis through secretion of the low-affinity epidermal growth factor receptor ligand amphiregulin (AREG), as observed in ischemic stroke mouse models. In contrast to other non-lymphoid tissues (e.g. intestine), the CNS is an immune-privileged compartment and largely devoid of Treg in steady state. However, a population of T cell receptor (TCR)αβ^+^CD4^+^Foxp3^+^ Treg has been found in the CNS of normal rats under non-inflammatory conditions, located mainly in the cerebral cortex. These Treg cells show an activated/memory phenotype characterized by the expression of inducible T cell costimulator (ICOS, also referred to as CD278), CD103, KLRG1, and CTLA-4. They express high levels of IL-10 and have the ability to suppress conventional T cells and restrain pro-inflammatory responses of microglia/macrophages in vitro [75].

Upon contact with resident CNS cells, naïve CD4+ T cells can convert to iTreg in vitro. Cytokine secretion and activation state of CNS myeloid cells directly influence T cell polarity. As found in vitro, IL-10 expression in microglia together with reduced expression of co-stimulatory molecules (CD40, CD80) promoted de novo induction of Foxp3 and the proliferation of pre-existing Treg in co-cultured CD4^+^ T cells [76]. These data indicate that mild CNS inflammation with low interferon (IFN)-γ levels induce a tolerogenic phenotype of microglia that favors expansion of Treg in the CNS. Similarly, co-incubation of astrocytes and lymphocytes induces T cells with suppressive properties, and supports the polarization of CD4^+^ T cells into Treg in vitro [77,78]. Astrocytes sustain Foxp3 expression in Treg through IL2/signal transducer and activator of transcription (STAT)5 signaling [75]. Moreover, interaction between neurons and T cells results in conversion of autoreactive T cells to Treg, which requires TGF-β [69]. Interestingly, adoptive transfer of Treg generated in glial and neuronal co-cultures suppresses encephalitogenic T cells and reduces the severity of EAE in rodents [69,76,78]. Although the extent to which peripheral T cells convert in the brain in autoimmune conditions is discussed controversially [79,80], these data indicate that Treg expansion is influenced by the crosstalk between lymphocytes and resident cells and the inflammatory environment in the CNS.

Besides their function in suppressing and terminating CNS inflammatory responses [81], Treg have been shown to promote neural tissue repair along with proliferation and differentiation of stem cells. Treg-secreted factors (Treg-conditioned media) enhanced the portion of myelin basic protein-expressing oligodendrocytes in murine mixed neuron-glia cultures and organotypic brain slices [82,83]. The Treg-derived growth regulatory protein CCN3 was shown to promote oligodendrocyte differentiation and myelination in vitro [83,84]. In addition, Treg-activated M2-type macrophages promote remyelination and differentiation of oligodendrocytes in the CNS [83,85]. Treg enhance neuronal stem cell proliferation in vitro and in the subventricular zone of ischemic mice in vivo. The beneficial effect of Treg on neurogenesis following brain injury is mediated through IL-10 signaling [86].

In contrast to their therapeutic effects in primarily autoimmune disorders and their potential to promote neural recovery, Treg reduce the protective antitumoral immunity and worsen the prognosis in neoplastic diseases of the CNS [87]. Opposing and probably disease phase-dependent effects of Treg have been described in animal models of neurodegenerative disorders and CNS injury [68,88,89]. Similarly, ambivalent functions of Treg can be observed in infectious disorders of the CNS (see below).

## 4. Regulatory T Cells in Virus Infection of the Nervous System

### 4.1. Role of Regulatory T Cells in Viral Encephalitis of Humans and their Animal Models

#### 4.1.1. Regulatory T Cells Show Neuroprotective and Antiviral Effects in Retroviral Encephalitis

Human immunodeficiency virus (HIV) causes a progressive loss of immune function, which increases the susceptibility to opportunistic infection and neoplasms. Infection of the brain is commonly associated with neuroinflammatory and neurodegenerative changes (Table 1), which leads to neurocognitive dysfunction in up to 50% of patients [90]. HIV-1-associated neurocognitive disorders (HAND) occur in advanced infections and encompass cognitive, motor, and behavioral abnormalities, ranging from subtle deficits to severe dementia resulting from encephalitis. The pathogenesis of HAND is not fully understood, but local virus replication and neuroinflammation are implicated in functional and structural damage to neural tissue. Microglial cells are one of the main reservoirs of latent HIV-1, and microglial activation is involved in the development of CNS lesions in HAND [91]. HIV-1-induced neuronal damage can be modeled in mice by intracranial injection of bone marrow-derived macrophages infected with a modified HIV-1 virus [92,93].

The role of Treg in the pathogenesis of systemic HIV infection remains controversial. Treg have been suggested to suppress retrovirus-specific immunity and inhibit viral clearance. In contrast, others describe a beneficial effect of Treg and their involvement in the inhibition of immune hyperactivation and control of viral load [35,94]. In a murine model of HIV encephalitis, protective effects of Treg on neuropathology and viral load were observed (Table 2). Herein, adoptively transferred green fluorescent protein (GFP)-labeled CD4^+^CD25^+^ Treg migrated across the blood–brain barrier and accumulated at sites of virus-induced inflammation in the murine brain [92]. The adoptive transfer of Treg into HIV-infected mice prevented neuronal damage. This was associated with reduced CD4^+^ T cell numbers, astrogliosis, microglial responses, tumor necrosis factor (TNF)-α expression and oxidative stress in the CNS. Moreover, transferred Treg enhanced the secretion of brain-derived (BDNF) and glial cell line-derived neurotrophic factor (GDNF) in infected rodents [93]. Using murine and human primary cell cultures, Treg have been shown to reduce iNOS and elevate arginase-1 protein levels in HIV-infected macrophages, indicating a switch from a neurotoxic M1 phenotype toward a M2 phenotype. These transformed myeloid cells were shown to exhibit neuroprotective properties in vitro, such as preserving dendrite morphology of cultured neurons [55].

Strikingly, Treg are also able to reduce viral loads in the brain of HIV-infected mice by increasing apoptosis of HIV-infected macrophages [93]. In vitro, Treg inhibited viral replication and release and actively killed HIV-infected macrophages by caspase-3 and perforin/granzyme-dependent pathways. The lethal effect on macrophages was markedly higher in infected compared to non-infected cells. In addition, co-cultivation with Treg induced proteomic changes in HIV-1-infected macrophages, characterized by upregulation of proteins related to antiviral immune responses, apoptosis, cell shape/motility, and metabolism, indicating that Treg function by a broad range of mechanisms to modulate the outcome of retroviral infection [55].

Collectively, these data indicate that although Treg may contribute to viral persistence in the periphery, CNS-infiltrating Treg have the potential to locally control HIV infection, resolve neuroinflammation, and promote neuronal survival in retroviral encephalitis.

#### 4.1.2. Regulatory T Cells Inhibit Antiviral Immunity and Facilitate Virus Latency and Spread, but also Protect from Excessive Immunopathology in Herpesvirus Infection

Infections with herpes simplex virus (HSV)-1 and -2 cause life-long virus latency and are the most common causes of sporadic fatal encephalitis in humans. Necrotizing encephalitis and myelitis (Table 1) can develop upon primary infection or reactivation of latent virus in ganglia [3]. Although HSV-1 is responsible for the majority of HSV encephalitis cases in adults and children, neonatal infection is often caused by HSV-2 [95]. Several studies have investigated the impact of Treg manipulation on HSV-infection outcome in mice. The results are partially contradictory, as positive and negative effects of Treg modulation are observed with respect to antiviral immunity (Table 2). Aside from the effects on viral load, Treg have been shown to dampen immunopathology in the CNS and eye in mouse models of HSV-infection.

##### Involvement of Regulatory T Cells in the Establishment and Reactivation of Latency

HSV infection triggers the expansion and activation Treg in lymphoid organs and at sites of inflammation in humans and mice, which putatively suppresses antiviral responses and contributes to immune evasion and the establishment of chronic infection or latency [96,97]. In the setting of ocular HSV-1 infection of mice, an inhibitory effect of Treg on antiviral immunity was demonstrated in experiments involving DT-mediated Foxp3 depletion. The model was characterized by an initial lytic phase with high-level virus replication at the infection site and within trigeminal ganglia, which is followed by virus clearance in the eye and establishment of latency in ganglia [96].

Treg co-localize with HSV-1 in the eye and trigeminal ganglia during acute and latent infection, resulting in suppression of cytotoxicity and antiviral cytokine production. Experiments involving DT-mediated Treg depletion and adoptive Treg transfer have demonstrated that this Treg influx provides critical assistance for the establishment of latency. Moreover, stress-induced reactivation of HSV-1 involves Treg expansion, which is presumably driven by glucocorticoid release [96]. Noteworthy, in contrast to mouse models of HSV-1 latency, virtually no Foxp3^+^ cells have been found in infected trigeminal ganglia in humans, despite the presence of CD8^+^ T cells, showing that human HSV-1 infection cannot be fully recapitulated by mouse models [108].

##### Involvement of Regulatory T Cells in Herpesvirus Spread to the Central Nervous System

Besides their role in latency and reactivation in ganglia, excessive Treg activity might also be involved in virus spread into the CNS and initiation of encephalitis, as shown in ocular, subcutaneous, and intranasal HSV infection.

In the ocular infection model, the Treg/T effector ratio was manipulated indirectly by influencing glucose metabolism. Limiting glucose utilization by the drug 2-deoxy-glycose reduces effector Th1 and innate immune cells but does not affect Treg frequencies in HSV-1-infected mice, leading to a skewed Treg/T effector ratio. Administration of the drug starting in the acute phase (from 0 dpi onwards) of ocular HSV-1 infection when replicating the virus is present in the eye, leads to reduced antiviral immunity and viral spread to the brain, resulting in fatal encephalitis. This effect appears to be disease phase-specific, as drug administration at later time-points (from 5 dpi), when viral load is low to absent in the eye, does not lead to fatal disease and shows protective effects on ocular immunopathology [109].

In the subcutaneous HSV-2 infection model, Treg suppression via anti-CD25 antibodies prior to infection enhanced early HSV-2-specific cytotoxicity and IFN-γ responses in neonatal and adult mice. In neonatal mice, this resulted in lower HSV-2 titers in the lymph nodes and CNS during acute infection, whereas no effects on viral load occurred in adults [98]. This supports the view that Treg responses of the immature immune system inhibit antiviral immunity, and can explain the increased susceptibility to lethal infections observed in human infants [98]. Moreover, Treg-depletion prior to infection using anti-CD25 antibodies or genetic Foxp3-ablation enhances innate and adaptive immune responses and increases IFN-γ production of CD8^+^ T cells in ganglia. Improved antiviral immune responses in Treg-depleted mice reduce HSV-2 infection in ganglia and decrease the severity of recurrent skin lesions from ganglionic spread [97].

Intranasal infection of mice with HSV-1 results in viral spread into the CNS along olfactory and trigeminal nerves. In this model, vitamin E deficiency was shown to increase Treg numbers in the periphery and the brain of infected mice, which was associated with decreased trafficking of IFN-γ-expressing CD8^+^T cells to the brain. Antibody-mediated Treg depletion prior to and during early infection (0-6 dpi) restores antigen-specific CD8^+^ T cell migration to the brain in vitamin E-deficient mice. However, probably due to a delayed onset of protective T cell responses, Treg-depletion fails to decrease the virus load and encephalic symptoms in this setting. Taken together, the timing of interplay between Treg and effector T cells during acute infection seems to be important for T cell trafficking to the brain, resolution of HSV-1 infection, and control of neurologic disease [99].

The described reports are in contrast with the findings described by Lund et al., which proposed an involvement of Treg in protection from virus spread to the CNS using genital HSV-2 infection [100]. In this model, the virus initially replicates in the vaginal mucosa and subsequently spreads into the CNS via retrograde trasport to the sacral ganglia, which results in fatal paralysis. In genital HSV-2 infection of mice, Foxp3-ablation prior to and during early infection led to a rapid fatal infection with an increased viral load in the spinal cord and neurologic deficits (hind limb paralysis), indicating that Treg facilitate protective immunity in the context of genital HSV-1 infection. Treg-deprived mice showed an increased chemokine ligand (CXCL)10, CCL2, CXCL9, and CXCL13 expression in innate immune cells in lymph nodes, but profoundly reduced INF production and immune cell numbers at the infection site. The pro-inflammatory chemokine secretion might cause an enhanced entry and retention of lymphocytes in lymphoid tissues, leading to a delayed recruitment of effector T cells, natural killer cells, and dendritic cells to the site of infection. The study indicates that Treg facilitate early protective responses to acute HSV infection by allowing a timely entry of immune cells into infected tissues [100].

The opposing effects of Treg manipulation on virus spread observed in the various murine models might be attributed to differences in infection route (e.g., ocular, cutaneous, nasal vs. genital) or other factors (e.g. genetic background and age of animals, Treg manipulation strategy, timing of modulation).

##### Involvement of Regulatory T Cells in the Suppression of Immunopathology

Besides their involvement in virus spread, studies in herpesvirus models have demonstrated a substantial involvement of Treg in suppressing excessive immunopathology in the CNS. In murine ocular HSV-1 infection, rapid antiviral treatment with aciclovir (≤ 2 dpi) protects from encephalitis by reducing CNS infection. However, when treatment is delayed (≥ 4 dpi), encephalitis escalates despite drug-induced virus control [110]. In a study conducted in mice receiving aciclovir at 4 days post HSV-1 infection, neuroprotective effects of Treg were demonstrated. Oral administration of *Bacteroides fragilis* polysaccharide A, an immunomodulatory bacterial component, induced CD39^+^CD73^+^ Treg and protected mice from fatal encephalitis following HSV-1 infection and delayed antiviral treatment. The bacterial symbiosis factor promoted IL-10 secretion by Treg and other cells, which restrained myeloid cell infiltration in the brain stem and pathogenic innate immune responses. This observation supports the idea that combinatorial treatment of an immunomodulatory compound and antiviral drug may be an effective treatment strategy for viral inflammatory diseases [110].

A protective role of Treg on neuroinflammation was also demonstrated in murine cytomegalovirus (MCMV) infection. MCMV encephalitis is a model for long-term neuroinflammation, characterized by immune cell infiltration from the periphery, activation of microglia, and secretion of pro-inflammatory cytokines, which persists even in the absence of detectable viral antigen. The inflammation is accompanied by local Treg accumulation, which is enhanced by IL-10-secreting B cells [111]. In the MCMV model, Treg prevented chronic neurologic dysfunction by controlling the development of reactive astro- and microgliosis in infected mice. It has been shown that Foxp3-depletion during acute MCMV infection enhances chronic glial cell responses, which heightens hippocampal damage and cognitive impairment in the post-encephalic phase [101,111]. Furthermore, Treg support the transition of effector T cells to tissue resident memory T cell in the CNS following MCMV infection. This CD103^+^CD8^+^ memory T cell population in MCMV-infected mice shows an enhanced cytotoxic activity (granzyme B release) upon antigen re-stimulation, which has the potential to control reinfection and reactivation of latent or persistent infection [102].

In summary, the majority of studies in HSV-1 or -2 infections show that Treg can negatively influence antiviral immunity, leading to systemic virus spread, CNS invasion, encephalitis, and lethality. However, dependent on the infection route, positive effects on local immune responses have also been demonstrated. This indicates that Treg might have different functions in distinct tissue sites. In scenarios with reduced viral replication (e.g., with concurrent antiviral treatment or in chronic MCMV infection), Treg seem to exert predominantly beneficial effects, as they ameliorate fatal immunopathology and aid in the establishment of immunologic memory.

#### 4.1.3. Regulatory T Cells Protect from Excessive Immunopathology in Acute Flaviviral Encephalitis, but Might Be Involved in the Establishment of Neuroinvasion

Flaviviruses transmitted by mosquitos or ticks are important causes of epidemic (meningo)encephalitis (Table 1), and are capable of causing serious morbidity and mortality [112]. West Nile virus (WNV) infection of humans is asymptomatic in approximately80% of cases and leads to a self-limiting febrile illness (West Nile fever) in the remaining 20%. Less than 1% of individuals develop a severe and potentially life-threatening neuroinvasive disease, which can result in long-term neurologic impairment in survivors [3,113]. The host factors responsible for development of neurologic disease are still largely elusive. On the one hand, an ineffective antiviral immune response can lead to neuroinvasion and virus persistence in the CNS, causing direct and indirect inflammation-induced damage. Long-term persistence of viral RNA has been reported following WNV infection and WNV-specific immunglobin M( IgM) antibodies have been detected up to 5 months post-infection in the cerebrospinal fluid (CSF) of patients with neurologic symptoms, indicating that virus persistence might be at least in part responsible for disease [114,115]. On the other hand, there is evidence that clinical disease is the result of immune-mediated damage caused by excessive immune responses, rather than neuroinvasion alone [116,117].

Japanese encephalitis virus (JEV) infection can cause an acute encephalitis resulting in mortality in up to 30% of cases, predominantly in infants. Approximately 50% of survivors develop serious neuropsychiatric sequelae, which are considered more fatal than WNV infection, given the fatality rate of 3%–5% [3]. Many aspects of the pathogenesis have been deciphered in rodent models of the disease. Following infection, JEV replicates in dendritic cells and macrophages in the periphery. CNS invasion is considered as the consequence of immune escape of the virus and crossing of the blood-brain barrier. Although JEV is able to kill neurons directly, it is also believed that an impaired blood-brain barrier and excessive secretion of pro-inflammatory cytokines, such as IL6 and TNF-a by infiltrating monocytes and activated microglia, markedly contribute to neuropathology and disease development [118,119].

Beneficial effects of elevated Treg numbers on the disease outcome have been suggested in humans and have been demonstrated in animal models of flavivirus encephalitis. However, animal experiments also indicate that Treg have negative effects on antiviral immunity and facilitate virus spread to the CNS.

##### Role of Regulatory T Cells in West Nile Virus Infection

In a cohort of 32 human patients with acute WNV infection, increased blood Treg levels were strongly correlated with asymptomatic infection [103]. In a different study, investigating 12 asymptomatic subjects and 12 patients with neuroinvasive disease, no differences were observed regarding overall Treg frequencies, but Treg from human patients with neurologic disease showed reduced CTLA-4 expression, suggestive of a reduced suppressive capacity. Moreover, these patients showed generally elevated, dysregulated, and atypically polarized T cell responses, which could be secondary to a diminished Treg function [116]. Therefore, a robust early Treg expansion might protect from symptomatic disease through dampening WNV-specific immune responses and associated immune-mediated damage [103,117]. The limitations of these studies are that samples were collected after infection of an unknown duration, and assessment of the pre-infection Treg levels was not possible. Moreover, the analysis of peripheral blood samples might not ideally reflect the situation in the CNS. Studies deciphering the connection of baseline Treg levels and infection outcome have been performed in animal models of WNV infection. 

Similar to humans, lower Treg levels can be found in C57BL/6 mice that succumb to acute WNV infection and increased frequencies of Treg expressing the activation and migration markers CD44, ICOS, and chemokine receptor (CXCR) 3 are associated with protection from clinically apparent neurologic disease [103,120]. Moreover, mice depleted of Treg prior to infection show increased neurologic disease symptoms and mortality following WNV infection [103]. Strikingly, although Treg-ablated mice exhibited higher frequencies of IFN-γ-producing, virus-specific T cells in the CNS during acute WNV infection, memory CD8^+^ T cell responses were impaired in later phases. Treg-mediated production of TGF-β resulted in an increase in CD103^+^CD8^+^ resident memory T cells in the brain at 60 dpi, indicating that Treg are able to maintain the brain resident memory population and potentiate antiviral immunity in WNV infection. Importantly, the effects on memory T cells were observed despite the fact that Treg depletion was only transient and performed prior to infection. The authors hypothesized that following infection, Treg might function to suppress a vigorous antiviral immune response to limit tissue damage at the cost of rapid virus clearance, but simultaneously protect the host from uncontrolled replication of persisting virus by aiding the development of a tissue resident memory T cell population [104]. This was supported by an experiment performed in mice on a broader genetic background. In order to identify factors that predispose to neuroinvasion and/or clinical disease, WNV infection was investigated using the collaborative cross (CC), composed of many different recombinant mouse lines with high levels of genetic variation, which models genetic diversity found in human populations. Similar to humans, individual CC strains show variable outcomes following WNV infection in terms of survival, clinical disease score, viral titer, and innate and adaptive immune responses in peripheral tissues and the CNS [121,122]. A correlation analysis of the infection outcome and immunophenotype suggests beneficial and detrimental effects of Treg, depending on the investigated parameter and time-point. Regarding the distribution and persistence of virus in CNS tissues, Treg seems to play a detrimental role. Reduced expression of activation markers on splenic Treg before infection are associated with peripheral virus restriction, suggesting that reduced suppressive activity of Treg under steady state conditions assists in preventing WNV neuroinvasion [120]. Moreover, an early expansion of splenic Treg with increased expression of CD73, CTLA-4, ICOS, glucocorticoid-induced tumor necrosis factor receptor TNFR-related protein (GITR), and CD44, as well as CNS migration markers such as CXCR3 and CD29, correlates with the manifestation of chronic WNV infection. An early brain infiltration of CD73^+^ Treg is associated with reduced cytolysis by natural killer cells and/or CD8^+^ T cells, likely allowing for viral persistence [122]. Additionally, elevated Treg levels are present in the murine brain during persistent WNV infection [123].

Taken together, prominent activation of the suppressive capacity and migratory ability of Treg during the early WNV infection and even prior to infection is associated with the establishment of chronic CNS infection. However, the connection between Treg and WNV persistence is based solely on correlation analyses thus far, and further experiments are needed to determine cause and effect. Treg depletion has been performed only in two experiments, and in one of them, viral load was not assessed [103], whereas the other showed no difference in viral RNA between Treg-depleted and non-depleted animals [104].

Importantly, not all CC mouse strains with neuroinfection develop clinical disease. In early neuroinvasive infection, high numbers of Treg expressing the activation markers CD44 and ICOS and the CNS migration marker CXCR3 correlate with protection from clinically apparent disease [120], which is in agreement with the findings described above [103]. Therefore, based on the current evidence, the beneficial effects of Treg seem to outweigh the possible impairment of antiviral immunity in clinical terms during established encephalitis. Analogous to the findings in WNV infection, a relative expansion of Treg compared to effector T cells is associated with reduced immunopathology and protection from severe disease in acute dengue virus infection in children [124].

##### Role of Regulatory T Cells in Japanese Encephalitis Virus Infection

In a mouse model of JEV infection, protective effects of CCR5^+^Treg were demonstrated. CCR5 ablation in JEV-infected mice exacerbated encephalitis symptoms and mortality, which was associated with reduced Treg responses and increased Th17 cell numbers in the brain. Adoptive transfer of CCR5^+^Treg into CCR5^−/−^ mice during development of encephalitis (3 dpi) reversed the phenomenon by increasing IL-10 and TGF-β expression in the brain. These data showed a beneficial role of Treg in dampening severe neuroinflammation following JEV infection, which depends on CCR5-mediated homing to the CNS. It is worth mentioning that CCR5^−/−^ animals with or without Treg-transfer have similar levels of virus in the CNS compared to JEV-infected wild-type animals, showing that neither CCR5 nor Treg have an impact on virus control and that the effects of the manipulations are unrelated to altered viral replication [105]. Interestingly, loss-of-function mutations in CCR5 are associated with increased disease severity, but not increased susceptibility in WNV-infected humans [125,126]. Whether this association is also related to CNS homing of Treg remains to be determined. Aside from CNS influx of peripherally generated or activated Treg, local generation of induced Treg might also be involved in ameliorating flaviviral encephalitis. Co-culture experiments reveal that neural stem/progenitor cells have the ability to convert encephalitogenic T cells isolated from JEV-infected mice into Treg [127].

In contrast to the neuroprotective effects observed during encephalitis, an involvement of excessive Treg activation in the periphery immediately following infection is suspected to contribute to immune escape of the virus. JEV is thought to manipulate the host’s immune system to evade the immune surveillance in the periphery and cross the blood–brain barrier. JEV infection led to disturbed maturation of human and mouse dendritic cells in vitro and ex vivo, which was also associated with elevated IL-10 production, programmed death-ligang 1 (PD-L1) expression, and expansion of the Treg population [128,129,130]. In contrast, in vitro dendritic cell infection with attenuated JEV (live vaccine) impaired the expansion of Treg, which might contribute to expansion of effector T cells and development of protective antiviral immunity following vaccination with the strain [131]. Therefore, similar to the situation in WNV infection, an initial induction of Treg putatively facilitates virus spread and neuroinvasion immediately following infection. Thus far, this has only been shown using in vitro or ex vivo culture of DCs. Further experiments are needed to determine the functional significance of JEV-induced Treg expansion in vivo. An association of Treg numbers and antiviral immunity has also been made in an animal experiment applying JEV-infected toll-like receptor (TLR)4-deficient mice. TLR4^−/−^ mice showed reduced viral CNS burden, lethality, and neuroinflammation, which is associated with enhanced type I IFN innate immunity and virus-specific CD4^+^ and CD8^+^ T cell responses and reduced Treg numbers. However, it remains unclear whether reduced Treg numbers in this setting are the cause or the effect of decreased CNS infection and inflammation, and further experiments are needed to demonstrate causality [132].

##### Role of Regulatory T Cells in *Zika virus* Infection

*Zika virus* infection during pregnancy causes developmental defects of the fetal CNS (Table 1). Similar to JEV, *Zika virus* infection induces indoleamine 2,3-dioxygenase 1 (IDO-1) expression in dendritic cells of human patients, which is thought to antagonize host antiviral immunity through Treg induction [4]. However, whether the early increase in peripheral Treg observed in *Zika virus*-infected mice affects protective immunity and contributes to neuroinvasive disease remains elusive [133].

In summary, there is direct evidence that Treg are capable of ameliorating immunopathology occurring in flaviviral encephalitis with favorable consequences for the host’s neurologic function and survival, once encephalitis is present. In vitro experiments and correlation analyses also suggest an involvement of Treg in facilitating immune escape and neuroinvasion of flaviviruses. Nonetheless, further experiments are needed to prove causality and functional relevance in vivo. From the perspective of the virus, enhancement of immunomodulatory mechanisms that facilitate virus spread but simultaneously ensures the survival of the host is certainly an advantage.

#### 4.1.4. Regulatory T Cells Inhibit Antiviral Immunity in Persistent Measles Virus Infection of the Central Nervous System

Mealses virus (MV) is a contagious respiratory pathogen that causes systemic disease and remains an important cause of child mortality [134]. Measles inclusion body encephalitis is a neurologic complication observed in immunocompromised individuals, which typically occurs within 1 year following acute MV infection. Subacute sclerotizing panencephalitis (SSPE) occurs on average 4–10 years following acute infection. Both CNS manifestations are characterized by extensive infection of neurons and oligodendrocytes with perivascular inflammation, degenerative changes, and gliosis (Table 1).

Elevated frequencies of circulating Treg can be observed in patients with acute measles, which might contribute to transient immunosuppression [135,136]. In addition, as observed in measles virus (MV)-infected rhesus macaques, Foxp3-expression in blood leukocytes correlated with slow viral clearance and prolonged infection [137]. Treg have been shown to influence the level of MV in the brain of persistently infected mice. In CD150-transgenic mice (which are susceptible to MV infection through the introduction of the human MV-receptor), expansion of splenic Treg and migration of these cells to the areas of viral replication in the brain can be observed following MV infection [138]. Treg survival and function are partly dependent on CD28 signaling. Employing a mouse model of persistent brain infection with recombinant MV, Treg expansion by superagonistic anti-CD28 antibodies (clone D665) during chronic MV infection induced transient immunosuppression and subsequent increase in virus replication and transneuronal spread in the mouse brain. Conversely, Foxp3-ablation in DEREG mice in a similar disease phase enhanced virus-specific CD8^+^ effector T cell responses in the brain and caused reduction in MV persistence [106,139]. Acid sphingomyelinase (Asm) deficiency increases the frequency and suppressive activity of Treg. MV infection of Asm-deficient mice led to reduced virus-specific CD8^+^ T cell responses and relatively enhanced Treg responses, resulting in massive brain infection. Similarly, Asm inhibition by amitriptyline, an antidepressant drug, increases the number of infected neurons in the mouse model persistent brain infection. The increase in viral brain load requires the presence of Treg because Asm inhibitor treatment shows no effect on MV infection in Treg-deprived mice [107]. Interestingly, in this study, Treg-depletion without Asm inhibition did not reproduce the effects on viral replication described in the study by Reuter et al. [106]. This could be due to differences in the protocol of Treg depletion (dose, time-point of DT administration) or due to different genetic backgrounds of used mice (C57BL/6 vs. C57BL/6.129). Because the described model of persistent MV infection did not produce clinically apparent disease and the neuropathologic sequelae were not investigated, the significance of differences in viral replication for neurologic function are unknown. Nevertheless, the reports indicated that manipulation of Treg in the periphery has consequences for the fate of MV infection in the brain. A disproportional increase in Treg compared to conventional T cells causes a poor control in MV infection, which may play a role in establishing persistent MV infection in subacute sclerotizing panencephalitis (SSPE). However, because decreased frequencies of circulating CD152^+^ Treg have been observed in SSPE patients, the role of peripheral and CNS infiltrating Treg in maintaining persistent MV infection in humans remains unclear [140].

In summary, excessive Treg functions increase viral CNS load in a mouse model of persistent MV infection.

#### 4.1.5. Other Viruses

An increased type I immune response with unchanged Foxp3^+^ cell levels was found in blood samples of influenza virus-infected children. In infected children with respiratory and neurologic complications (acute necrotizing encephalopathy), a high percentage of perforin- and IFN-γ-expressing CD4^+^ and CD8^+^ T cells, associated with low percentages of Treg were found, suggestive of a dysregulated antiviral type I immune response in complicated influenza virus infection [141]. Exuberant Th1 immune responses causing lethal meningoencephalitis in neonatal mice infected with Tacaribe arenavirus are associated with minimal IL-10 increase and absence of Treg [142].

### 4.2. Regulatory T Cells in Animal Models for Virus-Induced Demyelinating Disorders

Myelin damage in the CNS occurs following different neurotropic virus infections, such as JC virus (progressive multifocal leukoencephalopathy), MV (subacute sclerosing panencephalitis), and HIV infection. Although no clear causal relationship between MS and viral infection has been firmly established yet, viruses have been implicated in initiating or exacerbating MS symptoms. Important mouse models to study the pathogenesis of virus-induced demyelination are Theiler’s murine encephalomyelitis virus (TMEV) and neurotropic mouse hepatitis virus (MHV) infection. Infection with these viruses induces an acute encephalitis phase with virus replication within the CNS, followed by a phase characterized by demyelination, which occurs despite control of viral replication, and is driven by immunopathology or autoimmunity against myelin components. Because Treg-based therapies have been considered as an attractive therapeutic option for demyelinating diseases of humans, the effects of Treg manipulation in the context of virally-induced demyelination has been studied in both models (Table 3 and Table 4) [143,144].

#### 4.2.1. Theiler’s murine encephalomyelitis virus model

Infection of SJL mice with Theiler’s murine encephalomyelitis virus (TMEV) causes a biphasic disease with an acute and usually subclinical hippocampal infection, followed by a persistent infection of the spinal cord with myelin-specific autoimmunity and a clinically apparent demyelinating disease (TMEV-IDD), resembling chronic-progressive MS. By contrast, C57BL/6 mice eliminate the virus from the CNS during early infection by means of a robust antiviral CD8^+^ T cell response and develop no chronic disease [143].

A rapid systemic expansion and accelerated CNS infiltration of Treg were observed in SJL mice but not in C57BL/6 mice during early TMEV infection [145,146]. An unfavorable ratio of Treg to effector T cells during acute infection was proposed to inhibit antiviral cytotoxicity of TME-IDD-susceptible mouse strains, which contributes to TMEV persistence in the spinal cord. This hypothesis was tested in SJL mice by Treg-inactivation prior to TMEV infection using anti-CD25 and anti-GITR antibodies (Table 3). Both methods resulted in enhanced antiviral CD4^+^ and CD8^+^ T cell responses and TMEV-specific antibody production, which led to a reduced viral load in the CNS and delayed onset of neurologic disease [146]. Conversely, application of ex vivo-induced Treg (iTreg) prior to TMEV infection decreased CNS recruitment of leukocytes, resulting in increased virus replication and occurrence of clinical symptoms in the acute infection phase, which is usually asymptomatic. By contrast, Treg transfer during the chronic phase (3–4 weeks post infection) ameliorated demyelinating disease without affecting viral titers. These data showed that Treg have disease phase-dependent functions in the TME model, with detrimental effects on protective antiviral immunity during the initial phase and protective effects during chronic persistent infection, which are presumably mediated by suppression of virus-induced immunopathology [147]. Accordingly, beneficial effects of certain experimental treatments and pharmaceutical agents have been linked to increased Treg functions in the TMEV model. Dysbiosis induced by oral antibiotic administration prevented motor dysfunction and spinal axonopathy in TMEV-infected SJL mice, and the neuroprotective effect involved an enhanced CNS infiltration of CD39^+^ Treg [148]. Similarly, application of glatiramer acetate, an immunomodulatory drug used in MS treatment, improved clinical disease in TMEV-infected SJL mice with enhanced endogenous Treg responses and IL-10 production, but without affecting antiviral immunity [149]. 

In contrast to SJL mice, antibody-mediated Treg-depletion exhibited no impact on antiviral immunity in TMEV-IDD-resistant C57BL/6 mice [146]. Similarly, Foxp3-ablation in DEREG mice (C57BL/6 background) did not reduce the cerebral virus load, despite enhancing CNS recruitment of IFN-γ producing T cells [150]. Moreover, adaptive transfer of iTreg or expansion of endogenous Treg with IL-2 immune complexes failed to diminish virus clearance or to induce demyelination in C57BL/6 mice [147,151]. However, Treg expansion in combination with antibody-mediated CD8-depletion in C57BL/6 mice led to chronic TMEV infection with myelin loss and axonal damage in the spinal cord [151]. The combination of both manipulations resulted in a delayed recovery of depleted CD8^+^ T cells, which interfered with virus clearance. This finding indicates that, rather than the magnitude of Treg responses alone, the balance of Treg and CD8^+^ effector T cells instead is crucial to control virus replication upon acute infection. The lack of Treg-mediated effects on antiviral responses indicates that the function of Treg is overridden by the vigorous antiviral CD8^+^ T cell response in TME-IDD resistant C57BL/6 mice. 

In summary, detrimental effects of Treg on antiviral immunity depend on the genetic background of the animal, timing of manipulation, and the integrity of cytotoxic responses (Table 3).

The mechanisms, by which Treg exert their suppressive function in early TMEV-infection of SJL mice, remain uncertain. One of the most potent effectors secreted by Treg is IL-10, and elevated levels of the cytokine have been detected in brains of acutely infected SJL mice compared to lower levels in C57BL/6 mice [145]. In TMEV-infected SJL mice treated with antibodies blocking the IL-10 receptor, no positive effect on viral load in the brain or the spinal cord was observed during acute or chronic infection [152,153]. However, IL-10 blockade resulted in increased hippocampal damage during acute infection, which is usually only minimal or absent in SJL mice [152]. Thus, IL-10 secretion apparently represents a neuroprotective mechanism in acute TMEV-infection, whereas the suppressive effect on antiviral immunity is most likely mediated by other Treg effector functions.

#### 4.2.2. Coronavirus Model of Demyelination

The coronavirus model for demyelination is induced by experimental infection with neurotropic strains of mouse hepatitis virus (MHV). The disease course depends on the strain of MHV used to induce neurologic disease in susceptible strains of mice. Extremely virulent strains (e.g., JHM) cause acute encephalitis, whereas more attenuated strains (e.g., A59, J2.2v-1) are used to study acute disease and chronic demyelination. Manipulation of the Treg compartment has been performed in the acute as well as chronic disease model (Table 4) [144].

Following infection with neurovirulent JHM strain of MHV, insufficient Treg responses with dominant virus-specific CD4^+^ responses correlated with acute fatal encephalitis of mice. Treg seem to be critical for disease attenuation, as Treg transfer protects from fatal encephalitis following infection with highly neurovirulent strains. Moreover, Treg depletion prior to infection increased the mortality in mice infected with attenuated MHV JHM mutant, which usually results in non-lethal infection [154]. Similarly, Treg-depletion prior to acute infection with the attenuated MHV strain A59 led to increased numbers of apoptotic neurons in the brain but did not affect the CNS recruitment of virus-specific CD8^+^ and CD4^+^ T cells [155]. The studies indicates that in acute MHV encephalitis, Treg may help to limit immunopathology without inhibiting antiviral immunity [154,155]. Using tetramer techniques, virus-specific Treg populations expressing IL-10 and IFN-γ have been found in the CNS of mice infected with neuroattenuated MHV. These cells are thought to be thymus-derived natural Treg based on expression of the Helios transcription factor. They showed an activated state at the site of inflammation with high expression levels of CD69, ICOS, and CTLA-4, and had the ability to suppress MHV-specific effector T cells [70]. Using the neuroattenuated MHV strain (rJ2.2), virus-specific Treg have been shown to inhibit effector T cell expansion in lymph nodes and migration of pathogenic CD4^+^ T cells to the CNS. Adoptive transfer of MHV-specific Treg enhanced the survival rate of infected mice by reducing the frequencies of pathogenic CD4^+^ T cells and microglial responses in the brain in acute encephalitis [156].

A beneficial role of Treg has also been observed in chronic demyelinating disease caused by infection with attenuated MHV strains, although some differences have been observed depending on the time of Treg manipulation. Functional Treg inactivation by CD25-treatment prior to acute infection with neuroattenuated MHV strain increased demyelination during viral persistence, indicating that immunopathological processes resulting in myelin destruction are initiated at early stages and that they can be attenuated by Treg [157]. Moreover, the adoptive transfer of naïve Treg after initial effector T cell priming following infection with neuroattenuated MHV significantly reduced spinal cord demyelination and inflammation, as well as improving motor coordination deficits. Strikingly, transferred Treg did not compromise viral clearance or alter the percentage of virus-specific cells in the CNS of infected mice, showing a beneficial role of Treg also at a time when CNS infection has already been established. Transfer of Treg concomitant with MHV-specific T cells to infected recombination-activating gene (RAG)-deficient mice suggested that Treg reduce the migration of pathogenic CD4^+^ T cells to the brain by inhibiting the antigen presenting capacity of dendritic cells and pro-inflammatory cytokine and chemokine expression in the CNS-draining lymph nodes in MHV-infected mice [158,159]. In addition, Treg have been shown to inhibit the migration of myelin-specific T cells to the brain. The reduced CNS homing might be caused by Treg-mediated downregulation of CXCR3 expression on activated T cells in secondary lymphoid organs. The results indicated that Treg mainly attenuate autoreactive T cells during acute MHV infection but leave protective antiviral immunity intact [155].

By contrast, during persistent MHV infection, Treg seemto control myelin-specific T cell responses primarily in lymphoid organs but do not prevent autoimmune pathology in the CNS. Hence, Foxp3-ablation during the chronic phase does not affect inflammation, virus load, and autoimmune demyelination in the CNS. The disease phase-dependent effects of Treg-depletion with T cell retention in lymph nodes may be attributed to reduced effector T cell trafficking probably due to restored blood–brain barrier permeability in chronic infection. As observed in IL-27 receptor-deficient mice, IL-10-producing Tr1 cells infiltrated the CNS during chronic MHV infection and were critical for preventing autoimmunity and myelin loss at the site of persistent infection [160].

Intraspinal transplantation of neural precursor cells (NPC) derived from human embryonic stem cells reduced spinal cord inflammation and demyelination in MHV-infected mice. Clinical recovery in transplanted animals coincided with Treg infiltration in the spinal cord [161]. Similarly, transplantation of embryoid body-derived NPC transiently increased Treg numbers in CNS-draining lymph nodes, which correlated with remyelination and reduced neuroinflammation in MHV-infected mice [162]. Treg seem to be critical for white matter regeneration in MHV infection because antibody-mediated Treg-depletion increases myelin loss in transplanted mice. Co-culture experiments revealed that NPC increased Treg proliferation and induced conventional T cells to differentiate toward a Treg phenotype in vitro [161,162].

In summary, a therapeutic value of Treg enhancement for virus-induced demyelination and resulting clinical disease has been independently demonstrated in both mouse models, indicating that Treg-based therapies might be candidates for the treatment of human demyelinating diseases driven by autoimmunity. Moreover, the results are fairly consistent, regardless of the used model, virus strains, or method of Treg manipulation. However, adverse effects on viral load have been observed in the TMEV model. Here, a suppression of antiviral immunity occurred in the acute disease phase, characterized by high virus replication, whereas no effects are observed in chronic, low-level viral replication. Moreover, the effects were only observed in animals of a certain genetic background, which are highly prone to virus persistence by nature. TMEV infection has been performed in inbred mice with other genetic backgrounds (e.g., Balb/c, C3H, DBA/1, 129), and also recently in collaborative cross mice, resulting in a broad range of disease phenotypes and susceptibility to virus persistence [143,163]. However, the studies did not investigate the impact of Treg manipulation on infection outcome. In the coronavirus model, Treg manipulation did not affect virus control in the CNS. Nevertheless, all experiments have been performed in animals of the same C57BL/6 background and negative effects of Treg on MHV replication and persistence could occur in other mouse strains.

## 5. Conclusions and Outlook

The evidence collected by immunophenotyping of human samples with neurotropic infections suggests a connection between the magnitude and quality of Treg activity and the clinical outcome. In addition, immunomodulatory approaches including Treg-enhancement strategies represent attractive therapeutic methods for treating autoimmune neurologic diseases. Due to several limitations of studies in human subjects, animal models of viral CNS diseases have been employed to elucidate the relevance of certain immunophenotypes and their involvement in the pathogensis of infectious diseases. In the described studies, positive and negative effects of Treg-manipulation have been delineated in rodents, depending on the pathogen, infection route, disease phase, timing and method of intervention, genetic background of the host, and integrity of other components of the immune response. Moreover, the large body of data obtained in experimental settings clearly show multifaceted functions of the Treg population, broadening the view of Treg as purely immunosuppressive cells.

Elevated Treg frequencies and/or activation levels in humans are suspected to suppress antiviral immune responses and contribute to virus spread, CNS invasion, and establishment of virus persistence or latency. Indeed, animal models of ocular, intranasal and cutaneous HSV infection and the TMEV model for virus-induced demyelination confirmed the inhibitory effects of Treg on virus control. Interestingly, findings in both models indicated that the impact of Treg enhancement or depletion is highly dependent on the timing of the intervention. Although excessive Treg functions interfere with virus elimination and local containment in very early phases of infection, Treg appear to have negligible effects on virus control at later stages in these models. A similar situation is suspected in flaviviral encephalitis, as elevated Treg responses pre-infection correlate with virus spread and neuroinvasion, whereas high Treg numbers post-infection protect mice from fatal encephalitis.

In contrast to this, in mice infected with recombinant MV, the magnitude of Treg responses significantly influenced viral load in the persistently infected brain. Apparently, the qualitative and quantitative contribution of the Treg compartment to host defenses also depends on the infectious agent. Hence, clinical application of Treg-enhancing strategies bears the risk of exacerbating persistent or dormant infections or making the patient more susceptible to infections occurring in the course of treatment. This risk could potentially be reduced by the use of antigen-specific Treg recognizing self-epitopes, which are currently being developed [35].

In contrast to the mentioned findings, studies of experimental CNS neuroinfection have also revealed that Treg can contribute to virus control, for instance, by eliminating infected macrophages, as observed in the HIV-1 encephalitis model. Furthermore, the data generated in murine WNV and MCMV infection clearly showed that Treg are substantially involved in the establishment of tissue resident memory T cell populations in the CNS. Such antiviral functions of Treg have not been investigated in the majority of infectious models.

An undoubtedly and clinically highly relevant beneficial effect of Treg infiltration into the infected CNS is the suppression of excessive inflammatory responses and resulting protection of irreplaceable neuronal populations. Treg-mediated neuroprotective effects have been demonstrated in mouse models of retro-, herpes-, and flaviviral encephalitis. Treg-enhancing strategies further showed clear beneficial effects in viral models of chronic demyelinating diseases. Interestingly, in many cases of persistent CNS infections with low-level virus replication, elevated Treg levels did not seem to negatively influence viral burdens, suggesting that Treg somehow are able to modulate detrimental inflammation while ensuring sufficient virus control. The mode by which Treg can differentiate harmful and protective immune reactions remains enigmatic. It has been recently proposed that epigenetic instability of the Foxp3 locus could be a mechanism by which Treg differentiate between self- and non-self-antigens. According to this theory, Treg equipped with high affinity T cell receptors (TCR) for foreign antigens possibly occur due to the lack of negative selection in the thymus. Upon encounter of these antigens in infection, the strong TCR signal combined with the effects of pro-inflammatory cytokines at the infection site could destabilize Foxp3 expression, rendering Treg powerless or even converting them to effector T cells. Whether this phenomenon indeed occurs in vivo is still subject of debate because of contradicting results obtained in different fate-mapping experiments [164].

Lastly, the findings summarized in this review also highlight the influence of genetic background on immune system interactions. The best example is demonstrated in the TMEV model of demyelination, as manipulation of the Treg compartment has produced markedly different results in mice on the SJL and C57BL/6 background. The majority of animal experiments in the field of infectious diseases are conducted in standard inbred mouse strains with well-characterized genetic and phenotypic traits, resulting in predictable and reproducible results. However, these experiments do not allow for a generalization of statements regarding the safety of immunomodulation because they are not transferable to a population level. The development of collaborative cross (CC) mice facilitates the extension of infection models to a broader genetic background while maintaining reproducibility. It would be interesting to see how Treg manipulation influences disease outcomes in distinct CC strains. However, specific targeting of Treg is currently only feasible in Foxp3^DTR^ transgenic mice, which are only available on inbred backgrounds.

An open question regarding Treg involved in the pathogenesis of viral CNS infection is their origin. For a long time, it was assumed that peripheral Treg infiltrate neural tissues upon injury or disease. Some animal experiments used adoptive transfer of GFP-labeled Treg isolated from the periphery of donor mice, showing that Treg migrate from the periphery to the CNS upon neuroinflammation and exert local effects (e.g., [93,105]). In other experiments, the origin of Treg present in the infected CNS has not been investigated. Recently, distinct resident Treg populations have been described in various tissues, including the CNS. These tissue Treg are currently gaining increasing attention for their unique, site-specific properties and involvement in tissue regeneration [34,57,58].

## Figures and Tables

**Table 1:** Pathology of selected human virus infections of the central nervous system.

Group	Virus Family	Virus Name	Disease Name	Pathologic Findings in the Central Nervous System (Gross Findings; Histologic Findings)	References
RNA viruses	*Flaviviridae*	Zika virus	Congenital Zika syndrome	Microcephaly, ventriculomegaly; mononuclear infiltrates, gliosis, calcification, neuronal necrosis	[4,5,6,7,8]
		West Nile virus	West Nile encephalitis	Mononuclear infiltrates, gliosis, neuronal necrosis, neuronophagia, occasionally demyelination	[9]
		Japanese encephalitis virus	Japanese encephalitis	Cerebral congestion and edema; mononuclear infiltrates, gliosis, necrosis, hemorrhages, neuronophagia	[10]
	*Retroviridae*	Human immuno-deficiency virus (HIV)	HIV encephalitis	Mononuclear infiltrates, multinucleated giant cells, gliosis, neuronal loss, spongy myelinopathy, demyelination, vascular damage	[11]
	*Orthomyxoviridae*	Influenza virus	Influenza-associated acute encephalopathy	Cerebral edema and hemorrhage; neuronal apoptosis, necrosis, gliosis, occasionally mononuclear infiltrates	[12,13,14]
	*Paramyxoviridae*	Measles virus	Measles inclusion body encephalitis	Gliosis, intranuclear and cytoplasmic inclusion bodies in neurons and glial cells, mild mononuclear infiltration	[15,16,17]
			Subacute sclerosing panencephalitis	Cortical atrophy; mononuclear infiltrates, gliosis, neuronal necrosis and neuronophagia, intranuclear inclusion bodies (Cowdry type A) in neurons and oligodendrocytes, demyelination	[15,16,17]
		Nipah virus, Hendra virus	Henipavirus encephalitis	Mononuclear infiltrates, vasculitis and thrombosis with infarctions, neuronophagia, gliosis, endothelial syncytia with inclusion bodies	[18]
		Mumps virus	Mumps encephalitis	Mononuclear infiltrates, demyelination, gliosis, neuronal degeneration, hemorrhage, hyaline thrombi	[19]
	*Rhabdoviridae*	Rabies virus	Rabies	Mononuclear infiltrates, gliosis, neuronal necrosis, neuronophagia, cytoplasmic inclusion bodies (Negri bodies) in neurons	[20]
	*Bornaviridae*	Variegated squirrel 1 bornavirus	Borna virus-associated encephalitis	Mononuclear infiltrates, gliosis, edema, necrosis, neuronal necrosis, neuronophagia	[21,22]
	*Togaviridae*	Eastern equine encephalitis virus	Eastern equine encephalitis	Mononuclear infiltrates, gliosis, infarcts with hemorrhages, myelin pallor and Purkinje cell loss in cerebellum	[23]
	*Bunyaviridae*	La Crosse virus	La Crosse encephalitis	Mononuclear infiltrates, gliosis	[24]
DNA viruses	*Herpesviridae*	Herpes simplex virus	Herpes simplex encephalitis	Mononuclear infiltrates, hemorrhages, necrosis, intranuclear inclusion bodies (Cowdry type A) in neurons and glial cells	[25]
		Epstein–Barr virus (EBV)	EBV-associated encephalitis/vasculitis	Mononuclear infiltrates, vascular fibrinoid necrosis, hemorrhage, occasionally demyelination	[26,27,28]
		Varicella-zoster virus (VZV)	VZV encephalitis	Vasculopathy, vascular fibrinoid necrosis and thrombosis, necrosis, hemorrhagic infarcts, demyelination, intranuclear inclusion bodies (Cowdry type A) in glial and ependymal cells	[29,30]

**Table 2:** Role of regulatory T cells (Treg) in animal models of neurotropic virus infections, determined by direct Treg manipulation.

Model	Genetic Background of Mice	Method of Regulatory T Cell Manipulation	Timeframe	Effects on Antiviral Immunity	Effects on Immunopathology	References
*Retroviridae*						
HIV-1 encephalitis model	C57BL/6	Adoptive Treg transfer; Treg co-culture in vitro	*Acute infection* (Treg transfer: 1 dpi, analysis: 7 dpi)	Beneficial:Treg reduce viral replication and release, and destroy HIV-1-infected macrophages via caspase-3 and granzyme/perforin pathways	Beneficial: In vivo: Treg protect from neuronal loss, increase neurotrophic factor production, and reduce neuroinflammation In vitro: Treg induce proteomic changes in HIV-infected macrophages and transform them from M1 to M2 phenotype	[55,92,93]
*Herpesviridae*						
Ocular HSV-1 infection	BalB/c	DT-mediated Foxp3 ablation with or w/o adoptive Treg transfer	*Acute infection* (depletion: 4–6 dpi, analysis: 28 dpi) and *latent infection* (depletion: 26–27 dpi, analysis: 36 dpi)	Detrimental: *Acute phase:* Treg facilitate establishment of latency in trigeminl ganglia *Latent phase:* Treg are involved in stress-induced reactivation of latent infection	n.d.	[96]
Subcutaneous HSV-2 infection	C57BL/6	Antibody (CD25)-mediated Treg depletion or DT-mediated Foxp3 ablation	*Acute infection*(Treg depletion: -3 dpi, analysis: until 4 dpi)	Detrimental: Treg inhibit virus-specific CD4^+^ and CD8^+^ T cell responses, leading to increased viral load in the CNS of neonatal mice	n.d.	[97,98]
Intranasal HSV-1 infection	BALB/c	Vitamin E-deficient diet; antibody (CD25) mediated Treg depletion	*Acute infection*(diet: 4 weeks prior to infection; Treg depletion: -2 and 6 dpi, analysis: until 9 dpi)	Detrimental: Increased peripheral and CNS Treg numbers in vitamin E-deficient mice are associated with reduced trafficking of virus-specific CD8^+^T cells and increased viral load in the CNS	n.d.	[99]
Genital HSV-2 infection	C57BL/6	DT-mediated Foxp3 ablation with or w/o Treg transfer	*Acute infection* (Treg depletion: -2, 0, 3 dpi, analysis: úntil 12 dpi)	Beneficial: Treg limit initial replication and virus spread into the CNS by promoting entry of immune cells into the infection site	n.d.	[100]
Intracerebro-ventricular MCMV infection	C57BL/6	DT-mediated Foxp3 ablation	*Acute-chronic infection* (Treg depletion: -1, 1, 4 dpi, analysis: until 30 or 40 dpi)	Beneficial: Treg promote long-term immunity by supporting transition of effector T cells to tissue resident memory T cells	Beneficial: Treg reduce T cell numbers in acute encephalitis and supress microgliosis, astrogliosis, MHC class II expression, hippocampal neurotoxicity, and cognitive impairment in post-encephalitic phase	[101,102]
*Flaviviridae*						
Subcutaneous WNV infection	C57BL/6	DT-mediated Foxp3 ablation	*Acute infection* (Treg depletion: -1, 0 dpi, analysis: until 20 or 60 dpi)	No effect on viral load in acute infection; Treg limit effector T cell and inflammatory cytokine responses in acute encephalitis, but increase numbers of potentially protective memory T cells at later stages	Beneficial: Treg reduce morbidity and mortality in acute WNV encephalitis, presumably by reducing immunopathology	[103,104]
Intraperitoneal JEV infection	C57BL/6	CCR5^-/-^ mice with or w/o CCR5^+^ Treg or CCR^+^ Treg transfer	*Acute infection* (Treg tranfer: 3 dpi, analysis: until 15 dpi)	No effect	CCR5-mediated CNS homing of IL-10- and TGF-β-producing Treg reduces neuro-inflammation	[105]
*Paramyxoviridae*						
Intracerebral infection with recombinant MV	C57BL/6	Treg expansion by superagonistic CD28-antibodies; DT-mediated Foxp3 ablation	*Persistent infection* (Treg expansion: 14, 21 dpi, Treg depletion: 17–20 dpi, analysis: 28 dpi)	Detrimental: Treg inhibit virus-specific CD8^+^ T cell responses leading to increased virus replication in the persistently infected CNS	n.d.	[106]
Intracerebral infection with recombinant MV	C57BL/6, B6.129	Asm deficiency/blockade with or w/o concurrent DT-mediated Foxp3 ablation	*Persistent infection* (Asm blockade with or w/o Treg depletion: 21–26 dpi, analysis: 28 dpi)	Detrimental: Deficiency or inhibition of Asm leads to an elevated Treg to T effector ratio and results in increased virus replication (effect is Treg-dependent); no effect on viral load of Treg-depletion alone	n.d.	[107]

Abbreviations: Asm: acid sphingomyelinase; CNS: central nervous system; dpi: days post infection; DT: diphtheria toxin; Foxp3: forkhead box protein P3; HIV: human immunodeficiency virus; HSV: herpes simplex virus; IL: interleukin; JEV: Japanese encephalitis virus; MCMV: murine cytomegalovirus; MHC: major histocompatibility complex; MV: measles virus; n.d.: not determined; IL: Interleukin; TGF: transforming growth factor; Treg: regulatory T cell; WNV: West Nile virus.

**Table 3:** Effects of regulatory T cell manipulation on viral load and disease in Theiler’s murine encephalomyelitis virus infection.

Virus Strain	Mouse Strain	Treg ⇩ or ⇧	Time of Treg Manipulation*	Method	Viral Load	Disease	Reference
BeAn	SJL/J	⇩	Pre-infection	Antibody–mediated depletion (anti-CD25 or -GITR)	⇩	Delayed chronic demyelination	[146]
DA	SJL/J	⇧	Pre-infection	Adoptive transfer of ex vivo generated iTreg	⇧	Acute disease ⇧	[147]
DA	SJL/J	⇧	Chronic infection	Adoptive transfer of ex vivo generated iTreg	No effect	Chronic demyelination ⇩	[147]
DA	SJL/J	⇧**	Acute-chronic infection	Glatiramer acetate**	No effect	Chronic demyelination ⇩	[149]
DA	SJL/J	⇧**	Chronic infection	Oral antibiotics**	Not investigated	Chronic demyelination ⇩	[148]
BeAn	C57BL/6	⇩	Pre-infection	Antibody-mediated depletion (anti CD25)	No effect	No effect	[146]
BeAn	C57BL/6	⇩	Pre-infection	DT-mediated depletion in DEREG mice	No effect	No effect	[150]
DA	C57BL/6	⇧	Pre-infection	Adoptive transfer of ex vivo generated iTreg	No effect	No effect	[147]
BeAn	C57BL/6	⇧	Pre-infection	Expansion by IL-2C	No effect	No effect	[151]
BeAn	C57BL/6	Treg⇧ and CD8⇩	Pre-infection	Expansion by IL-2C, antibody-mediated CD8-depletion	⇧	Chronic demyelination ⇩	[151]

⇧ Increase or ⇩ decrease of regulatory T cell (Treg) numbers, viral load, or disease. * Time of Treg manipulation refers to the disease phase (pre-infection: before or at the day of intracranial infection; acute infection: 1–2 weeks post infection; chronic infection: 3–4 weeks post infection). ** Indirect effect on Treg. Abbreviations: DEREG: depletion of regulatory T cell mice (C57BL/6 background); GITR: glucocorticoid-induced tumor necrosis factor-receptor (TNFR)-related protein; IL2C: interleukin (IL) 2 immune complexes consisting of recombinant IL-2 and anti-IL-2-antibodies; iTreg: induced Treg.

**Table 4:** Effects of regulatory T cell manipulation on viral load and disease severity in acute and chronic infection with neurotropic mouse hepatitis virus.

Virus Strain	Mouse Strain	Treg ⇩ or ⇧	Time of Treg Manipulation*	Method	Viral Load	Disease Severity	Reference
Effects on acute encephalitis							
A59 (low virulence)	C57BL/6	⇩	Pre-infection-acute infection	DT-mediated depletion in DEREG mice	No effect	Encephalitis ⇧	[155]
rJ.M_Y135Q_ (low virulence)	C57BL/6	⇩	Pre-infection	Antibody–mediated depletion (anti-CD25)	No effect	Encephalitis ⇧ mortality ⇧	[154]
rJ (high virulence)	C57BL/6	⇧	Acute infection	Adoptive transfer of bulk Treg	No effect	Encephalitis ⇩ mortality ⇩	[154]
rJ.2.2 (low virulence)	C57BL/6	⇧	Pre-infection	Adoptive transfer of bulk or virus-specific Treg	No effect	Encephalitis ⇩ mortality ⇩	[156]
Effects on chronic demyelinating disease							
J2.2v-1 (low virulence)	C57BL/6	⇩	Pre-infection -acute infection	Antibody–mediated depletion (anti-CD25)	No effect	Demyelination ⇧	[157]
J2.2v-1, rJ2.2 (low virulence)	C57BL/6RAG1^-/-^	⇧	Acute infection	Adoptive transfer of bulk Treg	No effect	Demyelination ⇩	[159]
J2.2v-1 (low virulence)	C57BL/6	⇩	Transition from acute to chronic infection	DT-mediated depletion in DEREG mice	Virus RNA⇩ in CLN, no effect in CNS	No effect	[160]
J2.2v-1 (low virulence)	C57BL/6	⇧**	Transition from acute to chronic infection	Stem cell transfer**, antibody–mediated Treg depletion (anti-CD25)	No effect	Demyelination ⇩ remyelination ⇧	[161,162]

⇧ Increase or ⇩ decrease in regulatory T cell (Treg) numbers, viral load, or disease. * Time of Treg manipulation refers to the disease phase (pre-infection: before or at the day of intracranial infection; acute infection: first week post infection; chronic infection: from 14 days post infection). ** Indirect effect on Treg. Abbreviations: CLN: cervical lymph node; CNS: central nervous system; DEREG: depletion of regulatory T cell mice (C57BL/6 background); RAG1: recombination-activating gene 1 knockout mice (C57BL/6 background).

## Abbreviations

AMP	adenosine monophosphate
AREG	amphiregulin
Asm	acid sphingomyelinase
ATP	adenosine triphosphate
BDNF	brain-derived neurotrophic factor
CC	collaborative cross
CCL, CXCL	chemokine ligand
CCN3	cellular network communication factor 3
CCR, CXCR	chemokine receptor
CD	cluster of differentiation
CLN	cervical lymph node
CNS	central nervous system
CSF	cerebrospinal fluid
CTLA-4	cytotoxic T-lymphocyte-associated Protein 4
DC	dendritic cell
DEREG	depletion of regulatory T cells
DTR	diphtheria toxin receptor
EAE	experimental autoimmune encephalomyelitis
EBV	Epstein Barr virus
Foxp3	forkhead box protein P3
GDNF	glial cell line-derived neurotrophic factor
GFP	green fluorescent protein
GITR	glucocorticoid-induced TNFR-related protein
HAND	HIV-1 associated neurocognitive disorder
HIV	human immunodeficiency virus
HSV	herpes simplex virus
ICOS	inducible T cell costimulator
IDO-1	indoleamine 2,3-dioxygenase 1
IFN	interferon
IgM	immunglobulin M
IL	interleukin
IL-2C	IL-2-anti-IL-2-antibody complexes
iNOS	inducible nitric oxide synthase
IPEX	immune dysregulation, polyendocrinopathy, enteropathy, X-linked syndrome
iTreg	induced regulatory T cell
JEV	Japanese encephalitis virus
KLRG1	killer cell lectin-like receptor subfamily G member 1
LAG3	lymphocyte activation gene 3
MCMV	murine cytomegalovirus encephalitis
MHC	major histocompatibility complex
MHV	mouse hepatitis virus
MS	multiple sclerosis
MV	measles virus
NMDA	N-methyl-D-aspartate
NPC	neural precursor cells
nTreg	natural regulatory T cell
PD-L1	programmed death-ligand 1
RAG	recombination-activating gene
SSPE	subacute sclerotizing panencephalitis
STAT	signal transducer and activator of transcription
Tconv	conventional T cell
TCR	T cell receptor
TGF	transforming growth factor
TLR	toll-like receptor
TMEV	Theiler’s murine encephalomyelitis virus
TMEV-IDD	Theiler’s murine encephalomyelitis virus-induced demyelinating disease
TNF	tumor necrosis factor
TNFR	tumor necrosis factor receptor
Tr1	type 1 regulatory T cell
Treg	regulatory T cell
VZV	Varizella-zoster virus
WNV	West Nile virus
